# Increased Expression of Inactive Rhomboid Protein 2 in Circulating Monocytes after Acute Myocardial Infarction

**DOI:** 10.1007/s12265-024-10519-5

**Published:** 2024-05-14

**Authors:** Phillip van Dijck, Carmen Hannemann, Henryk Dreger, Verena Stangl, Karl Stangl, Antje Ludwig, Bernd Hewing

**Affiliations:** 1 Department of Cardiology, Angiology and Intensive Care Medicine, Campus Mitte, Deutsches Herzzentrum der Charité, Charitéplatz 1, 10117 Berlin, Germany; 2 DZHK (German Centre for Cardiovascular Research), Partner Site Berlin, Germany; 3 Division of Cardiology, Department of Medicine, New York University School of Medicine, New York, NY USA; 4 Berlin Institute of Health (BIH), 10178 Berlin, Germany; 5 Zentrum Für Kardiologie, Kardiologische Gemeinschaftspraxis, Muenster, Germany; 6 Present Address: Department of Cardiology III - Adult Congenital and Valvular Heart Disease, University Hospital Muenster, Muenster, Germany; 7 Department of Cardiology, Angiology and Intensive Care Medicine, Campus Virchow Klinikum, Deutsches Herzzentrum der Charité, Berlin, Germany; 8 Structural Heart Interventions Program (SHIP), Deutsches Herzzentrum der Charité, Berlin, Germany

**Keywords:** Myocardial infarction, TNF-alpha, iRhom2, Inflammation, Ventricular remodeling, Heart failure

## Abstract

Increased TNF-α levels following acute myocardial infarction (AMI) contribute to impaired recovery of myocardial function. Interaction of inactive rhomboid protein 2 (iRhom2) with TNF-α converting enzyme (TACE) is required for TNF-α shedding from immune cells. We hypothesized that iRhom2 expression increases in circulating monocytes following AMI. Transcript levels of iRhom2, TACE and TNF-α were evaluated by quantitative real-time PCR in isolated monocytes of 50 AMI patients at admission (d1) and 3 days (d3) after. We observed a significant increase in levels of iRhom2 mRNA expression in monocytes between d1-3, while TNF-α and TACE mRNA expression remained unchanged. At d3, iRhom2 mRNA expression positively correlated with levels of intermediate monocytes or serum TNF-α, and negatively with LV systolic function. iRhom2 may contribute to regulation of post-infarction inflammation and is associated with LV dysfunction following AMI. iRhom2 modulation should be evaluated as a potential therapeutic strategy to attenuate cardiac remodeling following AMI.

## Introduction

Acute myocardial infarction (AMI) is a leading cause of death worldwide [1]. Patients suffering a non-fatal AMI are exposed to elevated risk for short- and long-term complications including development of heart failure, ventricular arrhythmias, or recurrent AMI. Heart failure represents an important determinant for prognosis and survival in AMI patients [2]. The pathogenesis underlying heart failure development after AMI is closely related to inflammatory processes [3, 4]. Myocardial cell necrosis following AMI activates innate immunity and triggers a strong local as well as systemic inflammatory reaction that plays a central role in myocardial infarct healing but also in the development of adverse cardiac remodeling if dysregulated, excessive or prolonged [5]. The inflammatory cytokine tumor necrosis factor-alpha (TNF-α) is detectable in the infarcted myocardium already during the early phase of AMI [6] and elevated serum TNF-α levels have consistently been described in patients with AMI [7]. Animal studies have shown that TNF-α is involved in impaired recovery of myocardial function following AMI [8–10] whereas different animal models of genetic or pharmacological TNF-α inhibition have shown promise in attenuating important parameters of adverse cardiac remodeling [11–13]. Controlled clinical trials addressing the potential of anti-TNF-α therapy in AMI are not available to date. TNF-α is mainly expressed by immune cells (in particular by monocytes and macrophages) as a precursor transmembrane protein located at the cell surface. Transmembrane TNF-α is cleaved by transmembrane metalloprotease TNF-α converting enzyme (TACE; ADAM17) to be released from the cell surface as soluble TNF-α (TNF-α shedding) [14]. Soluble TNF-α exerts its biological effects by binding to TNF receptor type 1 and 2 (TNFR1 and 2) [15]. TACE maturation in immune cells depends on inactive rhomboid protein 2 (iRhom2; RHBDF2) [16, 17]. Genetic deficiency or knock-down of iRhom2 prevents the activation of TACE and subsequently diminishes TNF-α shedding from immune cells. TACE maturation in non-hematopoietic cells remains unaltered as co-expression of closely related iRhom1 compensates for the absence of iRhom2 in these cells [18]. Up to date, the impact of the iRhom2/TACE/TNF-α-axis in AMI is unknown. In a previous study, we have shown that iRhom2-deficiency attenuates atherosclerosis development in low density lipoprotein receptor deficient mice [19]. Given the importance of iRhom2 in the regulation of TACE and TNF-α in immune cells, we aimed to investigate the gene-expression of iRhom2 in patients following AMI. We hypothesized that iRhom2 expression increases in circulating monocytes of patients with AMI, providing first evidence that iRhom2 is involved in the regulation of postischemic inflammation following AMI.

## Methods

### Study Design and Population

The study protocol was approved by the Ethics Committee of the Charité-Universitätsmedizin Berlin (EA1/246/14). The study complied with the Declaration of Helsinki. Written informed consent was obtained from each study participant. From June 2015-February 2017, patients with AMI (n = 50), were recruited at the Charité-Universitätsmedizin Berlin, Germany. The AMI group comprised patients with ST-segment Elevation (STEMI) and Non-ST-segment Elevation Myocardial Infarction (NSTEMI) as defined by the guidelines of the European Society of Cardiology [20, 21]. AMI patients were recruited within 24 h after initial hospital admission with completed cardiac catheterization. Inclusion criteria comprised: age ≥ 18 years and diagnosis of AMI (as defined above). Exclusion criteria comprised the need for cardiac resuscitation, fever, acute infection/sepsis, rheumatic or non-rheumatic autoimmune diseases, current immunosuppressive therapy, severe acute or chronic renal failure (GFR < 30 mL/min/1.73 m^2^), malignant diseases, stroke, or surgery within 30 days. AMI patients were evaluated by medical history, clinical examination, and blood collection at study inclusion and at day 3 after study inclusion. Medical history was assessed by interviews and information from medical records. Functional class was assessed according to the New York Heart Association (NYHA) classification. Furthermore, standard transthoracic echocardiographic exams were performed within the first days after hospital admission (mean 2.4 ± 1.6 days after hospital admission) and were digitally stored for offline analysis (TOMTEC-ARENA). Left ventricular ejection fraction (LVEF) was measured using the biplane Simpson’s method as previously described [22]. For patients where image quality was not sufficient for assessment of biplane LVEF, visually estimated LVEF was used. LVEF was categorized into I: less than 30%, severely abnormal; II: 30% to 39%, moderately to severely abnormal; III: 40% to 49%, moderately abnormal; IV: 50% to 53%, mildly abnormal; V: > 54%, normal.

### Blood Sampling and Standard Laboratory Parameters

Peripheral blood samples were collected from cubital veins. Standard laboratory parameters including differential blood count, C-reactive protein (CRP), creatine kinase (CK), interleukin-6 (IL-6), N-terminal pro-B-type natriuretic peptide (NT-proBNP), TNF-α, and high sensitivity troponin T (hs-Troponin) were obtained by established assays in the hospital’s laboratory.

### Monocyte Isolation

Peripheral blood mononuclear cells (PBMC) were obtained by Ficoll density gradient centrifugation (Ficoll-PaqueTM PLUS, GE Healthcare) of 30 ml peripheral blood collected in EDTA tubes. On average, 30*10^7^ PBMCs were isolated per sample. Subsequently, monocytes were isolated by negative selection using the human Pan Monocyte Isolation Kit (Miltenyi Biotec; MACS isolation) according to the manufacturer’s instructions. Purity of enriched monocytes was confirmed by flow cytometry as detailed below. Approximately 7*10^7^ monocytes were isolated per sample by MACS isolation.

### Flow Cytometry

200 µL eluate of MACS isolated cell fraction was stained with monoclonal antibodies (CD86 PE [B7-2; Biolegend], CD14 PB [M5E2; Biolegend], CD16 APC [3G8; Biolegend], CD11b PECy7 [ICRF44; Biolegend]) for 15 min followed by repeated (twice) washing with 2 ml phosphate-buffered saline (PBS) solution. After centrifugation and removal of supernatant, cells were resuspended in PBS and run on a flow cytometer (CyAn™ ADP Analyzer; Beckman Coulter, Inc.). Data was analyzed using Summit™ 4.4 software applying the following gating strategy: first, peripheral blood mononuclear cells (PBMCs) were chosen and plotted on a CD86 histogram; monocytes were identified as CD86 + cells and purity of enriched monocytes was defined as percentage of monocytes compared to all cells detected. On average, purity of enriched monocytes following MACS isolation was 90%. Secondly, monocytes were divided into three subsets: CD14 +  + CD16- (classical), CD14 +  + CD16 + (intermediate) and CD14-CD16 +  + (non-classical) according to the surface expression pattern of CD14 and CD16 [23]. To differentiate between intermediate and non-classical monocytes a straight vertical line was drawn to the left of the CD14 staining of the classical monocytes in the CD14 and CD16 dot plot as described previously [24]. Analyses of flow cytometry data was performed by two blinded observers; results represent averaged values from both analyses.

### Quantitative Real-Time RT-PCR

RNA from MACS sorted monocytes was isolated using RNeasy Mini Kit (Qiagen) according to the manufacturer’s instructions. 500 ng of total RNA was reversed-transcribed using the High-Capacity cDNA Reverse Transcription Kit (Applied Biosystems). TaqMan assays (Applied Biosystems) were used to quantify the expression of iRhom2 (RHBF2; Assay ID: Hs01078106_m1, catalog #4331182), TACE (Assay ID: Hs01041915_m1, catalog, #4331182), and TNF-α (Assay ID: Hs01113624_g1, catalog #4331182). RPL19 (Assay ID: Hs02338565_gH, catalog #4331182) was used as housekeeping gene. Expression of the target gene relative to housekeeping gene expression was calculated as the difference between the threshold values for the two genes. Relative expression levels were normalized to a young and healthy reference group (Healthy). Twenty-five young and apparently healthy volunteers (mean age 22.8 years, range 19–27 years; 44% female) without known cardiovascular disease were therefore recruited.

### Statistical Analysis

Results are generally expressed as arithmetic mean ± SD for normally distributed data and median with interquartile ranges (IQR = 25th-75th percentile) for non-normally distributed data; Shapiro–Wilk-test was used for testing for normal distribution of the data. Categorical data are presented as absolute numbers with respective percentages. For normally distributed data the Student’s t-test was performed for either paired (one-sample t-test) or unpaired (independent two-sample t-test) observations. For non-normally distributed data the Mann–Whitney-U-test was used for comparison of two independent groups, while the Wilcoxon signed-rank test was used for comparison of paired observations. Pearson’s or Spearman’s correlation coefficient, where appropriate, were calculated for correlation analyses between variables. Statistical analyses were performed using SPSS 25.0 (SPSS Inc.) software; *p* < 0.05 was considered statistically significant.

## Results

### Clinical Characteristics

Clinical characteristics of AMI patients at study inclusion are shown in Table 1. The mean age of the study population was 55 years with 28% of the study population being female. Based on electrocardiographic findings 34 patients were diagnosed with STEMI and 16 patients with NSTEMI. For 82% of the participants this AMI was the primary manifestation of coronary artery disease. Fourty-six AMI patients received drug eluting stent implantation during cardiac catheterization, none of the patients underwent coronary artery bypass grafting (CABG) or required percutaneous left ventricular assist device. The average LVEF of AMI patients was 50.4 ± 9.3%. Patients were treated with guideline directed medical therapy during hospitalization [20, 25]. Five patients had pre-existing statin therapy before hospital admission. Standard laboratory parameters are shown in Table 2. At study entry, all patients had elevated troponin levels meeting the diagnostic criteria for myocardial infarction. None of the participants had laboratory signs of relevant renal dysfunction on day 1.

**Table 1:** Clinical characteristics of AMI patients at study inclusion (day 1)

Characteristic		
Age in years, mean (range)		55 (33–85)
Sex, *n* (%)	male	36 (72.0)
	female	14 (28.0)
BMI (kg/m^2^)		28.6 ± 5.8
NSTEMI, *n* (%)		16 (32.0)
STEMI, *n* (%)		34 (68.0)
CV risk factors		
IDDM, *n* (%)		3 (6.0)
NIDDM, *n* (%)		5 (10.0)
Active smoking, *n* (%)		26 (52.0)
Hypercholesterolemia, *n* (%)		29 (58.0)
Hypertension, *n* (%)		29 (58.0)
Family history of CVD, *n* (%)		11 (22.0)
CVD history		
Previous ACS, *n* (%)		6 (12.0)
Previous PCI, *n* (%)		8 (16.0)
Previous CABG, *n* (%)		0
Previous TIA, *n* (%)		1 (2.0)
Previous Stroke, *n* (%)		0
PAD, *n* (%)		0
Catheterization data		
Initial diagnosis CAD, *n* (%)		41 (82.0)
CAD-1 vessel, *n* (%)		22 (44.0)
CAD-2 vessel, *n* (%)		11 (22.0)
CAD-3 vessel, *n* (%)		17 (34.0)
Main vessel disease, *n* (%)		2 (4.0)
PTCA, *n* (%)		49 (98.0)
Stent, *n* (%)		46 (92.0)

Data are generally expressed as mean ± SD, or absolute numbers and respective percentages

*BMI* body mass index, *NSTEMI* non-ST-elevation myocardial infarction, *STEMI* ST-elevation myocardial infarction, *CV* cardiovascular, *CVD* CV disease,* IDDM* insulin dependent diabetes mellitus, *NIDDM* non insulin dependent diabetes mellitus, *ACS* acute coronary syndrome, *PCI* percutaneous coronary intervention, *CABG* coronary artery bypass grafting, *TIA* transient ischemic attack, *PAD* peripheral artery disease, *CAD* coronary artery disease, *PTCA* percutaneous transluminal coronary angioplasty; stent, only drug eluting stents were used

**Table 2:** Laboratory parameters

Parameter	AMI day 1	AMI day 3	*p*-value AMI day 1 vs. day 3
Troponin-T high sensitive, ng/L	1052.0 (419.0–4403.0)	1233.5 (439.0–2068.0)	**0.001**
Creatine kinase, U/L	593 (265–1242)	181.00 (127–311)	** < 0.001**
NT-Pro-BNP, ng/L	642.0 (212.0–1359.0)	569.5 (308.0–1411.0)	**0.004**
Creatinine, mg/dl	0.84 (0.77–0.93)	0.86 (0.77–1.01)	**0.021**

Data are expressed as median with interquartile ranges. Statistical analysis was performed using the Wilcoxon signed-rank test. *NT-proBNP* N-terminal pro-B-type natriuretic peptide

### Serum TNF-α Levels

**Fig. 1:**
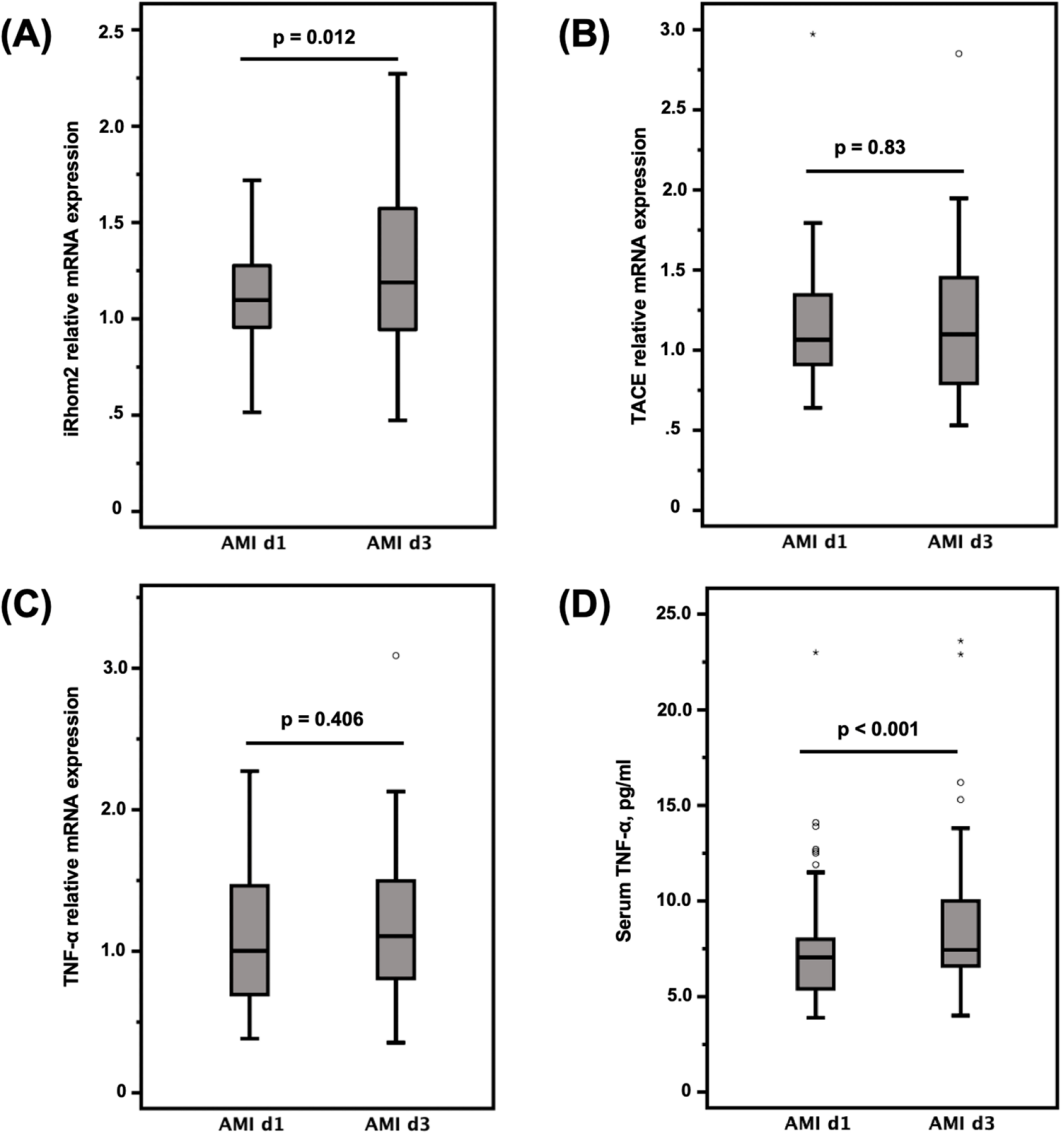
Relative mRNA expression of iRhom2 (**A**), TACE (**B**), TNF-α (**C**) in monocytes in AMI patients on day 1 compared to day 3 after hospital admission. (**D**) Serum TNF-α levels of AMI-patients on day 1 compared to day 3. Statistical analysis for comparison between day 1 and day 3 was performed using the student’s t-test for normally distributed data and the Wilcoxon signed-rank test for non-normally distributed data, mild outliers (1.5*IQR—3.0*IQR); *, extreme outliers (> 3,0*IQR); AMI, acute myocardial infarction; d1 or 3, day 1 or 3

AMI patients showed a slight but significant increase of serum TNF-α levels from day 1 to day 3 after AMI (p < 0.001) (Fig. 1 and Table 3). Furthermore, CRP and IL-6 serum levels were increased and changed significantly from day 1 to day 3 (Table 3), all indicating a systemic inflammatory response following AMI.

**Table 3:** Blood levels of inflammatory markers

Parameter	AMI day 1	AMI day 3	*p*-value, AMI day 1 vs. day 3
TNF-α, pg/ml	7.05 (5.4–8.0)	7.45 (6.6–10.0)	** < 0.001**
CRP, mg/L	5.3 (2.0–12.3)	19.5 (11.6–41.8)	** < 0.001**
IL-6, ng/L	16.95 (10.5–31.1)	11.95 (7.4–19.2)	**0.007**

Data are expressed as median with interquartile ranges. Statistical analysis was performed using the Wilcoxon signed-rank test

*TNF* tumor necrosis factor, *CRP* C-reactive protein, *IL-6* Interleukin 6

### Circulating Monocytes Subsets

The absolute count of circulating monocytes in patients with AMI did not change significantly from day 1 to day 3 (Table 4). Relative levels of circulating intermediate monocytes increased significantly from day 1 to day 3 after AMI (Table 4). In contrast, relative levels of circulating classical monocytes decreased, while levels of circulating non-classical monocytes remained unaltered from day 1 to day 3 after AMI (Table 4).

**Table 4:** Absolute monocyte count and relative levels of circulating monocyte subsets

	AMI day 1	AMI day 3	*p*-value, AMI day 1 vs. day 3
Monocytes, /μl	889.1 ± 0.29	908.8 ± 0.28	0.68
Classical Monocytes (%)	87.7 (82.5–91.8)	85.3 (80.8–88.8)	**0.025**
Intermediate Monocytes (%)	5.07 (3.4–7.4)	8.50 (6.3–10.2)	** < 0.001**
Non-Classical Monocytes (%)	3.72 (2.3–5.6)	3.93 (2.1–5.7)	0.214

Data are expressed as mean ± SD or as median with interquartile ranges. Statistical analysis was performed using a paired Student's t-test for normally distributed data and the Wilcoxon signed-rank test for non-normally distributed data

### iRhom2, TACE, and TNF-α mRNA Expression in Circulating Monocytes

iRhom2 mRNA expression levels in circulating monocytes of AMI patients increased significantly (by 14%) from day 1 to day 3 after AMI **(**Fig. 1 and Table 5). No significant changes in mRNA expression levels of TACE and TNF-α in circulating monocytes of AMI patients were observed between day 1 and day 3 after AMI (Fig. 1 and Table 5). We observed a strong correlation between iRhom2 and TACE mRNA expression in circulating monocytes on day 3 after AMI (r = 0.82, *p* < 0.001). Furthermore, iRhom2 mRNA expression in circulating monocytes correlated moderately with serum TNF-α levels (r = 0.33, *p* = 0.019) and with relative levels of circulating intermediate monocytes (r = 0.37, *p* = 0.009) on day 3. Serum TNF-α levels correlated with relative levels of circulating intermediate monocytes on day 1 (r = 0.40, *p* = 0.004) and day 3 (r = 0.29, *p* = 0.039).

**Table 5:**
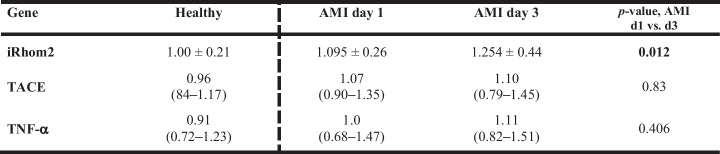
Relative mRNA expression of iRhom2, TACE und TNF-α in circulating monocytes

Data are expressed as mean ± SD or as median with interquartile ranges. Statistical analysis between AMI day 1 and day 3 was performed using a paired Student's t-test for normally distributed data and the Wilcoxon signed-rank test for non-normally distributed data. Expression levels were normalized to a young and healthy reference group (Healthy)

*iRhom2* inactive rhomboid protein 2, *TACE* TNF-α converting enzyme, *TNF* tumor necrosis factor

### Association of iRhom2 mRNA Expression in Circulating Monocytes and LV Systolic Function

Blood levels of NTproBNP, Troponin T, and TNF-α are associated with LV systolic dysfunction following AMI [26]. According to our present observation that iRhom2 expression in monocytes increases in the course of AMI correlation analyses with LV systolic function obtained from transthoracic echocardiography were performed. We found that LV ejection fraction (categorized LVEF) negatively correlated with iRhom2 mRNA expression in circulating monocytes on day 3 after AMI, with the difference (Δ) of iRhom2 mRNA expression in circulating monocytes between day 1 versus day 3, and with serum TNF-α levels on day 3 (Table 6).

**Table 6:** Correlations with left ventricular ejection fraction (LVEF)

Parameter	LVEF (categorized)	
	Spearman rho	*p*-value
iRhom2 mRNA expression, day 1	−0.26	0.070
iRhom2 mRNA expression, day 3	−0.34	**0.025**
Δ iRhom2 mRNA expression, day 1 vs. 3	−0.36	**0.010**
Serum TNF-α, day 1	−0.19	0.186
Serum TNF-α, day 3	−0.36	**0.010**
NT-proBNP, day 1	−0.59	** < 0.001**
NT-proBNP, day 3	−0.57	** < 0.001**
Troponin-T high sensitivity, day 1	−0.47	**0.001**
Troponin-T high sensitivity, day 3	−0.45	**0.001**

*iRhom2* inactive rhomboid protein 2, *TNF* tumor necrosis factor, *NT-proBNP* N-terminal pro-B-type natriuretic peptide. Statistical analysis was performed using the Spearman's rank correlation coefficient

## Discussion

In the present pilot study, we investigated the iRhom2/TACE/TNF-α-axis as a potentially important regulator of postischemic inflammation in patients with AMI. Similar to previous studies, we observed a significant increase of serum TNF-α levels within the first 3 days following AMI as part of a systemic inflammatory response (further indicated by the increase of CRP between day 1 and 3) [27, 28]. The interaction of iRhom2 with TACE is essential for proper shedding of TNF-α from the cell surface of immune cells [16]. Accordingly we found that mRNA expression levels of iRhom2 in circulating monocytes increased in parallel to serum TNF-α levels following AMI. In contrast, TACE and TNF-α mRNA expression in circulating monocytes remained unchanged indicating a central regulatory role of iRhom2 in TNF-α response following AMI. This was further supported by a significant correlation between serum TNF-α levels and iRhom2 expression levels in circulation monocytes on day 3 after AMI. Among monocyte subsets intermediate monocytes are considered the major source of serum TNF-α [29]. In fact, we observed a significant correlation between relative levels of circulating intermediate monocytes not only with serum TNF-α levels but also with iRhom2 mRNA expression levels on day 3 following AMI. Taken together these findings strongly suggest a relevant regulatory role of iRhom2 in the augmented shedding of TNF-α from intermediate monocytes in the early phase of AMI.

Given the fact that excess levels of TNF-α are known to impair myocardial recovery and promote postischemic myocardial injury, the observed significant correlation between iRhom2 mRNA expression in circulating monocytes on day 3 following AMI with the extent of LV dysfunction in our patient population appears to be a consistent outcome. These results suggest that modulation of iRhom2 represents a promising novel target to reduce excess TNF-α secretion from immune cells after AMI to attenuate adverse cardiac remodeling.

Modulation of iRhom2 is considered a more distinguished strategy compared to non-selective TNF-α blockage as it presumably reduces cardiotoxic effects of soluble TNF-α (by reducing transmembrane TNF-α shedding selectively from immune cells) more selectively while preserving cardioprotective functions of the cytokine in its transmembrane form [19]. Soluble TNF-α primarily binds to TNFR1, which promotes inflammation and apoptosis [30]. In contrast, cardioprotective effects of TNFR2 such as tissue healing, angiogenesis, and anti-inflammatory processes (primarily mediated by transmembrane TNF-α) would be preserved [30, 31].

There are some limitations in the present study. First, due to the observational character of this pilot study the results remain descriptive. Secondly, expression analyses of iRhom2 and TACE were limited to mRNA levels. It is known that iRhom2 RNA and protein levels in macrophages increase following LPS stimulation [16, 32]. Furthermore, increasing iRhom2 mRNA expression levels in macrophages are followed by increases of soluble TNF-α levels, thus indicating a solid association between iRhom2 mRNA expression and TNF-α protein levels [16]. Thirdly, a longer time-course for the evaluation of the iRhom2/TACE/TNF-α-axis beyond day 3 following AMI was not performed due to the generally intended early hospital discharge of patients after AMI.

In conclusion, the present study suggests that iRhom2 contributes to the regulation of inflammation and is thereby associated with LV systolic dysfunction following AMI. Thus, iRhom2 modulation should be further evaluated as a potential therapeutic strategy in AMI patients to attenuate adverse cardiac remodeling.

## Data Availability

Data are available from the corresponding author BH upon reasonable request.

## Abbreviations

ACS: Acute coronary syndrome
AMI: Acute myocardial infarction
BMI: Body mass index
CABG: Coronary artery bypass grafting
CAD: Coronary artery disease
CV: Cardiovascular
CVD: CV disease
iRhom2: Inactive rhomboid protein 2
LVEF: Left ventricular ejection fraction
NSTEMI: Non-ST-elevation myocardial infarction
NT-proBNP: N-terminal pro-B-type natriuretic peptide
NYHA: New York Heart Association
PAD: Peripheral artery disease
PCI: Percutaneous coronary intervention
PTCA: Percutaneous transluminal coronary angioplasty
STEMI: ST-elevation myocardial infarction
TACE: TNF-α converting enzyme
TIA: Transient ischemic attack
TNF-α: Tumor necrosis factor-alpha
TNFR1 and 2: TNF receptor type 1 and 2

## Declarations

### Animal Subject

No animal studies were carried out by the authors for this article.
